# Laughter yoga as an enjoyable therapeutic approach for glycemic control in individuals with type 2 diabetes: A randomized controlled trial

**DOI:** 10.3389/fendo.2023.1148468

**Published:** 2023-03-31

**Authors:** Mayumi Hirosaki, Tetsuya Ohira, Yawei Wu, Eri Eguchi, Kokoro Shirai, Hironori Imano, Narumi Funakubo, Hitoshi Nishizawa, Naoto Katakami, Iichiro Shimomura, Hiroyasu Iso

**Affiliations:** ^1^ Department of Epidemiology, Fukushima Medical University School of Medicine, Fukushima, Japan; ^2^ Center for Southeast Asian Studies, Kyoto University, Kyoto, Japan; ^3^ Public Health, Department of Social Medicine, Graduate School of Medicine, Osaka University, Osaka, Japan; ^4^ Department of Public Health, Faculty of Medicine, Kindai University, Osaka, Japan; ^5^ Department of Metabolic Medicine, Graduate School of Medicine, Osaka University, Osaka, Japan

**Keywords:** type 2 diabetes, laughter, laughter yoga, glycemic control, self-care, positive affect

## Abstract

**Background:**

Laughter has been reported to have various health benefits. However, data on the long-term effects of laughter interventions on diabetes are limited. This study aimed to investigate whether laughter yoga can improve glycemic control among individuals with type 2 diabetes.

**Methods:**

In a single-center, randomized controlled trial, 42 participants with type 2 diabetes were randomly assigned to either the intervention or the control group. The intervention consisted of a 12-week laughter yoga program. Hemoglobin A1c (HbA1c), body weight, waist circumference, psychological factors, and sleep duration were evaluated at baseline and week 12.

**Results:**

Intention-to-treat analysis showed that participants in the laughter yoga group experienced significant improvements in HbA1c levels (between-group difference: −0.31%; 95% CI −0.54, −0.09) and positive affect scores (between-group difference: 0.62 points; 95% CI 0.003, 1.23). Sleep duration tended to increase in the laughter yoga group with a between-group difference of 0.4 hours (95% CI −0.05, 0.86; *P* = 0.080). The mean attendance rate for laughter yoga program was high (92.9%).

**Conclusions:**

A 12-week laughter yoga program is feasible for individuals with type 2 diabetes and improves glycemic control. These findings suggest that having fun could be a self-care intervention. Further studies with larger numbers of participants are warranted to better evaluate the effects of laughter yoga.

**Clinical trial registration:**

http://www.chinadrugtrials.org.cn, identifier UMIN000047164.

## 1 Introduction

Diabetes is a chronic condition that requires lifelong self-management for metabolic control, such as healthy eating, regular physical activity, and self-monitoring of the blood glucose levels, as well as taking medication as prescribed (1, 2). Previous studies have shown that good glycemic control maintenance is important for reducing the risk of diabetes-related complications and mortality (3–5). However, it has been reported that about 40-60% of individuals with type 2 diabetes have not achieved good glycemic control in all regions of the world (6). For example, about 60% of individuals with type 2 diabetes in Japan and about half of those in the US, have not achieved the HbA1c < 7% glycemic target (7, 8). Furthermore, a study has found that despite greater utilization of newly developed glucose-lowering medications, concurrent improvements in overall glycemic control were not shown (9). These findings suggest that existing therapeutic options may not be sufficient for maintaining good glycemic control and investigating new complementary therapeutic approaches would be needed.

Previous studies have also found that individuals with type 2 diabetes had poor adherence to exercise and a healthy diet (10, 11). It would be difficult to sustain lifestyle changes (12), and efforts toward lifestyle modifications have the potential to cause emotional distress. On the other hand, it has been suggested that positive affect experienced during health behaviors facilitates long-term adherence to the behaviors (13). Previous studies have shown that people are more likely to intend to engage in a health behavior and to actually engage in it, when the behavior is seen as enjoyable (14, 15). A systematic review has found that pleasant affect experienced during exercise predicts future physical activity (16). Thus, we expected that enjoyable activities, which boost positive psychological states, would be useful as self-care behavior in individuals with type 2 diabetes.

In this study, we focused on laughter as an enjoyable diabetes self-care intervention. Laughter has been demonstrated to have various health benefits, such as reducing stress (17), enhancing the activities of natural killer cells (17–19), suppressing allergic reactions (20), and inhibiting increases in postprandial blood glucose levels (21). Prospective cohort studies demonstrated that a low frequency of laughter is associated with increased risks of functional disabilities, all-cause mortality, and cardiovascular diseases (22, 23). Additionally, laughter interventions have a positive effect on depression and the well-being of older adults (24).

We previously reported that the combination of laughter and exercise decreased hemoglobin A1c (HbA1c) levels among older adults (25). In that study, we combined watching comedy programs with a general exercise program to increase the participants’ enjoyment and enhance their motivation to attend the intervention. However, making all participants laugh by watching the same comedy programs is difficult. Different individuals have different preferences. In contrast, self-induced laughter, including laughter yoga, which combines simulated laughter with deep breathing, does not require humorous stimuli (26). Additionally, a study that examined the potential benefits of laughter-inducing therapies has suggested that simulated laughter not caused by humor or other stimuli is more effective than spontaneous laughter triggered by humorous stimuli (27). Therefore, we conducted a laughter yoga intervention in this study.

Laughter yoga is now used in many countries (28). Several studies have shown that it improves depressive symptoms (29), decrease the levels of stress hormones (30, 31), and improves heart rate variability (32). Additionally, a study has reported that laughter yoga inhibits increases in postprandial blood glucose levels in individuals with type 2 diabetes (33). However, that study has shown the effect of a single 30-min session of laughter yoga only, and to the best of our knowledge, little is known about the long-term effects of laughter yoga on diabetes.

Therefore, this 12-week randomized controlled trial investigated the effects of laughter yoga on glycemic control and psychological well-being in individuals with type 2 diabetes.

## 2 Materials and methods

### 2.1 Study design and participants

This study was a single-center, two-group, randomized controlled trial designed to evaluate the effects of laughter yoga on individuals with type 2 diabetes. The intervention was conducted at Osaka University from October 2015 to December 2015. The study protocol was approved by the Scientific Ethics Committee of Fukushima Medical University (no. 2028). The Declaration of Helsinki was followed, and reporting in this article is aligned with the Consolidated Standards of Reporting Trials standards. All participants provided oral and written informed consent. This trial was registered at the University Hospital Medical Information Network Clinical Trials Registry (no. UMIN000047164).

Participants were recruited at the Diabetes Center of Osaka University Hospital, between August 2015 and September 2015. Three diabetologists evaluated their outpatients for study eligibility and approached patients meeting the eligibility criteria. Eligible patients received a flyer and the study was explained. Patients were recruited in order of their visit until the number of participants reached the target sample size. The inclusion criteria were outpatients aged 40 years or older with type 2 diabetes, HbA1c levels ranging from 6.1% to 7.9%, and changes in HbA1c levels < 1.0% during the last 3 months before baseline measurements. The glycemic control target is set at HbA1c <7.0% in Japan and modification of treatment (including intensive pharmacotherapy or insulin treatment) is needed when HbA1c level exceeded 8% (34). In addition, it would be difficult to determine whether the change in HbA1c level is due to the laughter yoga intervention or other factors in patients with originally large HbA1c fluctuations (unstable diabetes) before the laughter yoga intervention. Therefore, we included patients with HbA1c level changes of <1.0% during the last 3 months before baseline measurements (with stable treatment status). Patients with active coronary heart disease or stroke were excluded to reduce the risk of adverse events due to the intervention (e.g., a cardiac event during physical activity). Additionally, patients who could participate in light to moderate–intensity exercises were included, and those with other vascular complications and severe illness were excluded.

Before randomization, the participants were informed that similar laughter yoga sessions would be held after the study period for those who were allocated to the control group to reduce the reporting bias.

### 2.2 Intervention

All participants continued to receive standard therapy for diabetes as they received before the study began. The control group continued the standard therapy and the intervention group received the standard therapy plus the laughter yoga program. Standard therapy included taking oral hypoglycemic medications, receiving advice from the doctor in charge of dietary modifications and physical activity in accordance with the “Treatment Guide for Diabetes” in Japan (35). Three participants received insulin therapy (one participant in the intervention group and two in the control group); the other participants received oral hypoglycemic agents. The control group was instructed to spend the study period as usual.

The participants in the intervention group received laughter yoga program once a week during the first 4 weeks and then every other week during the last 8 weeks. In total, eight sessions over 12 weeks were provided. The duration of laughter yoga intervention in previous studies ranged from 4 weeks to 8 weeks (28). However, a systematic review assessing the effects of yoga intervention on cardiovascular disease risk factors reported that the effects were most prominent in randomized controlled trials with 12 weeks of intervention duration (36). Therefore, we considered 12 weeks of intervention as appropriate. Every session began with a lecture of approximately 30 min on laughter and health, followed by a 60-min laughter yoga session. The laughter yoga session was based on the standardized laughter yoga program and the mini-lecture was added. The purpose of the lecture was to relax the participants and create a friendly atmosphere before the laughter yoga. Laughter yoga sessions were group-based interventions and guided by certified laughter yoga trainers from the Japan Laughter Yoga Association. All sessions were delivered by the same laughter yoga trainers to unify the contents of the intervention.

Laughter yoga is a kind of exercise consisting of deep breathing and voluntary laughter in a sitting or standing position. Each laughter yoga session consisted of warm up exercises, deep-breathing exercise, laughter exercise, and calming activity. At the beginning, the participants were asked to clap their hands along with saying the phrase “Ho, Ho, Ha, Ha, Ha,” as a warm up exercises. Then, deep breathing with laughter were performed. Subsequently, the participants were asked to participate in voluntary laughing imaging in a variety of situations, including 5-min break. For example, when doing the “milkshake laughter,” participants were asked to imagine that they have a glass of milk in their right hand and a glass of their favorite fruit juice in their left hand. They pretended to pour the milk from one glass into the other and pour it back into the first glass (to mix them). Then, they pretended to drink the milkshake, with a laugh. Finally, the participants were asked to close their eyes and relax.

### 2.3 Outcomes

The primary outcome was changes in HbA1c levels from the baseline to the 12-week follow-up measurement. Exploratory outcomes included changes in body weight, waist circumference, body mass index (BMI), positive affect, negative affect, subjective stress, and sleep duration from baseline to the 12-week follow-up. Physical examination and self-administered questionnaire were assessed on weeks 0 and 12.

HbA1c levels were measured in capillary whole blood, collected by finger prick, using the COBAS b101 point-of-care system (Roche Diagnostics International Ltd, Rotkreuz, Switzerland). Body weights were measured using a UC-322 weighing scale (A&D Co. Ltd., Tokyo, Japan), and BMIs were calculated as follows: weight (kg)/height squared (m^2^). Waist circumference was measured halfway between the lower border of the ribs and the iliac crest using a measuring tape.

The following data were obtained *via* a self-administered questionnaire: height; lifestyle factors, such as smoking and drinking habits, sleep duration, and physical activity; and psychological factors. Positive and negative affects were assessed using the Japanese version of the 15-item Geriatric Depression Scale (GDS-15) (37). Most study participants were at least 65 years old. Thus, the GDS-15 was used for the assessment of psychological status. The GDS-15 is widely used to screen depression among older adults. Although the factor structure of the GDS-15 varies across study populations and language groups, a meta-analysis showed that the positive mood factor, including five items (Are you basically satisfied with your life? Are you in good spirits most of the time? Do you feel happy most of the time? Do you think it is wonderful to be alive now? and Do you feel full of energy)? is the most similar across languages (38). In this study, we used the 5 items as an indicator of positive affect and another 10 items as an indicator of negative affect. In a previous study, the Cronbach’s alpha value was 0.72 for the 5 positive affect items and 0.82 for the 10 negative affect items in community-dwelling older adults in Japan (39). All GDS-15 items were assessed in a “yes/no” format. We calculated the sum of the presence of the positive affect and that of the negative affect (yes = 1, no = 0), respectively. In this study, positive (0–5) and negative (0–10) affect summary scores were created. Subjective stress was assessed using a single item (“Do you feel stressed at work or in daily life?”) with four response options: very much = 4, quite a lot = 3, a little = 2, and not at all = 1. In addition, we took attendance in every session and calculated the attendance rate.

### 2.4 Sample size calculation

A previous study reporting that laughter can decrease HbA1c levels in older adults without diabetes (25) revealed a 0.19% difference in HbA1c changes between the intervention and control groups. A meta-analysis reported that low to moderate–intensity resistance exercises reduced HbA1c levels by 0.23% in individuals with type 2 diabetes (40). Therefore, we assumed a 0.2% difference in the mean decrease in HbA1c levels between the intervention and control groups (standard deviation of 0.2). A sample size of 44 participants (22 in each group) was sufficient to detect the difference of 0.2% between groups using a two-tailed t-test of the difference between means with 90% power and a 5% significant level. The required sample size was 48, considering a dropout rate of 10%.

### 2.5 Randomization

After baseline measurements were completed, the participants were stratified according to sex and randomly allocated to either the intervention (laughter yoga program and standard therapy) or control (standard therapy only) group in a 1:1 ratio according to a computer-generated sequence. All randomization was carried out by a researcher who was not involved with participant enrollment. Participants were informed of their group assignment after consent and baseline measurements. Outcome assessors were blinded to group allocation.

### 2.6 Statistical analysis

Data were analyzed according to an intention to treat principle, with the baseline value carried forward for missing data. The differences in baseline characteristics between the two groups were tested using independent samples *t* tests for continuous variables and chi-square tests for categorical variables. Mann-Whitney U tests were used when the continuous variables were not normally distributed. The changes in measurements between the baseline and the 12-week follow-up in both groups were compared using paired-samples *t* tests. Unadjusted differences of changes from baseline to the 12-week follow-up between the two groups were analyzed using the independent samples *t* tests. Differences in changes between the two groups adjusting for age, BMI and each dependent variable value at baseline were analyzed using analysis of covariance. As a further check, per-protocol analyses excluding dropouts were conducted. Attendance rates for laughter yoga program were calculated by dividing the number of sessions attended by the number of sessions prescribed (41). All analyses were conducted using Statistical Package for the Social Sciences (IBM Corporation, Armonk, NY, USA). *P*-values of less than 0.05 were used to denote statistical significance.

## 3 Results

**Figure 1:**
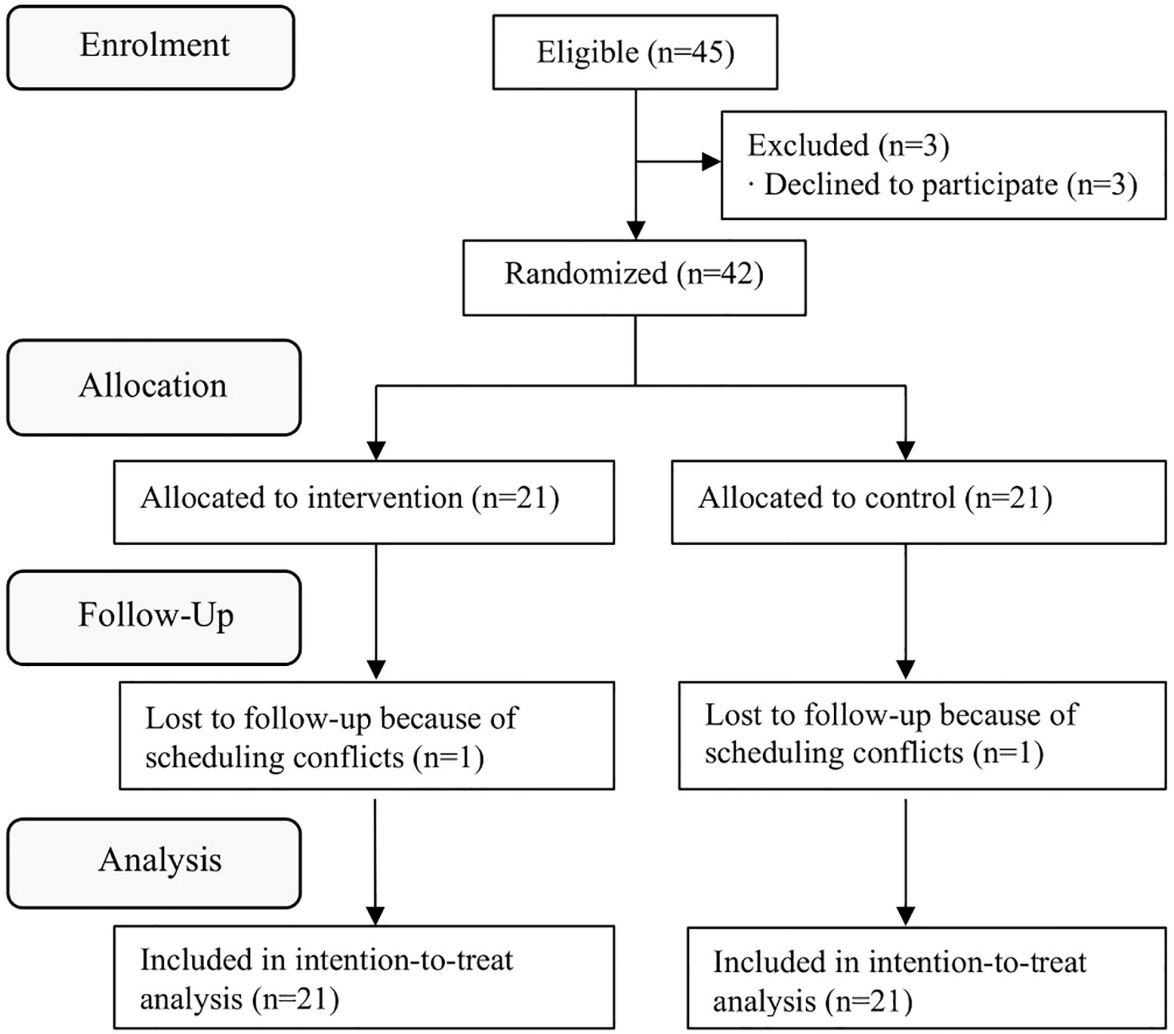
Consolidated Standards of Reporting Trials (CONSORT) diagram of study participants.

Forty-five eligible participants were enrolled in the study, and 42 agreed to participate in this study and underwent baseline measurements. Twenty-one participants were assigned to the laughter yoga group and 21 participants were assigned to the control group (**Figure 1**). One person in each group dropped out because of personal reasons. Both groups had high retention rates of 95%. The mean attendance rate for the laughter yoga program was 92.9%. No serious adverse events occurred. No changes in the content of the medical treatment occurred in either group during the study period.

The baseline characteristics of the 42 participants stratified by groups are shown in **Table 1**. No significant differences in baseline characteristics were observed between the two groups. The Cronbach’s alpha value was 0.70 for the positive affect items and 0.51 for the negative affect items.

**Table 1:** Baseline characteristics of the participants.

	Laughter yoga group	Control group	*p*-value
	(n = 21)	(n = 21)	
Age, years	71.8 (6.4)	70.6 (8.2)	0.641
Women, n (%)	14 (66.7)	14 (66.7)	1.000
Body weight, kg	58.1 (9.2)	62.2 (9.7)	0.165
BMI, kg/m^2^	23.4 (3.2)	24.7 (2.8)	0.156
Waist circumference, cm	87.5 (9.3)	91.9 (8.3)	0.116
HbA1c, %	7.07 (0.7)	7.19 (0.7)	0.580
Positive affect	3.4 (1.6)	3.9 (1.3)	0.454
Negative affect	2.1 (1.6)	2.0 (1.7)	0.745
Subjective stress	1.9 (0.7)	2.2 (0.9)	0.320
Sleep duration, hours	6.0 (0.9)	6.2 (1.3)	0.542
Exercise, ≥2 days/week, n (%)	14 (66.7)	10 (47.6)	0.350
Current alcohol drinking, n (%)	3 (14.3)	8 (38.1)	0.160
Current smoking, n (%)	0 (0)	0 (0)	1.000
Number of family members	3.2 (1.6)	3.0 (1.4)	0.731

BMI, body mass index; HbA1c, hemoglobin A1c. Data are mean (SD) unless otherwise indicated.

**Table 2** presents the changes from baseline to 12th week follow-up. The mean HbA1c levels changed from 7.07% to 6.82% in the laughter yoga group and from 7.19% to 7.26% in the control group. The unadjusted difference between the two groups was statistically significant (−0.31%; 95% confidence interval [CI]: −0.54% to −0.09%; *P* = 0.008). The adjusted difference (adjustment for age, BMI and HbA1c level at baseline) was also significant (*P* = 0.002). The positive affect score significantly increased in the laughter yoga group (between-group difference: 0.62 points; 95% CI 0.003 to 1.23; *P* = 0.049), although the adjusted between-group difference (adjustment for age, BMI and positive affect score at baseline) was not statistically significant (*P* = 0.139). A trend toward an increase in sleep duration was observed in the laughter yoga group with a between-group difference of 0.4 hours (unadjusted: 95% CI −0.05 to 0.86; *P* = 0.080), and the adjusted difference was marginally significant (*P* = 0.054). No significant differences in body weight, BMI, waist circumference, negative affect, and subjective stress were found between groups. Per-protocol analyses excluding dropouts and missing data in the follow-up questionnaire revealed a very similar pattern of results for all outcomes (**Table 3**).

**Table 2:** Physiological and psychological changes during the intervention in the laughter yoga and control groups.

	Baseline		12 weeks		Δ0-12 weeks	*p-*value^a^	Between-group difference (95% CI)	*p*-value^b^	*p*-value^c^
	Mean (SD)		Mean (SD)		(95% CI)				
HbA1c (%)									
Laughter yoga (n = 21)	7.07	(0.7)	6.82	(0.6)	−0.24 (−0.39, −0.09)	0.004	−0.31 (−0.54, −0.09)	0.008	0.002
Control (n = 21)	7.19	(0.7)	7.26	(0.7)	0.07 (−0.11, 0.24)	0.410			
Body weight (kg)									
Laughter yoga (n = 21)	58.1	(9.2)	58.2	(9.3)	0.15 (−0.23, 0.54)	0.421	−0.36 (−0.88, 0.16)	0.166	0.164
Control (n = 21)	62.2	(9.7)	62.7	(9.5)	0.51 (0.15, 0.88)	0.009			
BMI (kg/m^2^)									
Laughter yoga (n = 21)	23.4	(3.2)	23.5	(3.2)	0.06 (−0.10, 0.22)	0.447	−0.16 (−0.38, 0.06)	0.146	0.134
Control (n = 21)	24.7	(2.8)	25.0	(2.8)	0.22 (0.06, 0.37)	0.010			
Waist circumference (cm)									
Laughter yoga (n = 21)	87.5	(9.3)	86.7	(8.8)	−0.82 (−2.17, 0.52)	0.217	−1.04 (−3.07, 0.97)	0.302	0.141
Control (n = 21)	91.9	(8.3)	92.1	(8.2)	0.22 (−1.37, 1.82)	0.773			
Positive affect									
Laughter yoga (n = 21)	3.4	(1.6)	4.1	(1.2)	0.62 (0.11, 1.13)	0.020	0.62 (0.003, 1.23)	0.049	0.139
Control (n = 21)	3.9	(1.3)	3.9	(1.0)	0.00 (−0.38, 0.38)	1.000			
Negative affect									
Laughter yoga (n = 21)	2.1	(1.6)	2.2	(1.8)	0.05 (−0.54, 0.63)	0.867	−0.05 (−0.74, 0.64)	0.890	0.786
Control (n = 21)	2.0	(1.7)	2.1	(1.6)	0.10 (−0.31, 0.50)	0.629			
Subjective stress									
Laughter yoga (n = 21)	1.9	(0.7)	2.1	(1.0)	0.14 (−0.16, 0.44)	0.329	0.34 (−0.11, 0.77)	0.133	0.339
Control (n = 21)	2.2	(0.9)	2.0	(0.6)	−0.19 (−0.53, 0.15)	0.258			
Sleep duration (hours)									
Laughter yoga (n = 21)	6.0	(0.9)	6.3	(1.0)	0.33 (−0.04, 0.70)	0.074	0.40 (−0.05, 0.86)	0.080	0.054
Control (n = 21)	6.2	(1.3)	6.1	(1.4)	−0.07 (−0.40, 0.22)	0.614			

BMI, body mass index; HbA1c, hemoglobin A1c; SD, standard deviation; 95% CI, 95% confidence interval.

a P for comparing the difference in outcomes before and after the intervention using paired-samples t test.

b P for unadjusted between-group difference in changes in outcomes over 12 weeks using independent samples t test.

c P for adjusted between-group difference (adjustment for age, BMI and each dependent variable value at baseline) using analysis of covariance.

**Table 3:** Physiological and psychological changes during the intervention in the laughter yoga and control groups using per-protocol analyses.

	Baseline		12 weeks		Δ0-12 weeks	*p-*value^a^	Between-group difference (95% CI)	*p*-value^b^	*p*-value^c^
	Mean (SD)		Mean (SD)		(95% CI)				
HbA1c (%)									
Laughter yoga (n = 20)	7.12	(0.7)	6.86	(0.6)	−0.26 (−0.42, −0.09)	0.004	−0.33 (−0.57, −0.09)	0.008	0.003
Control (n = 20)	7.20	(0.7)	7.27	(0.7)	0.08 (−0.11, 0.26)	0.410			
Body weight (kg)									
Laughter yoga (n = 20)	58.3	(9.4)	58.5	(9.5)	0.16 (−0.25, 0.57)	0.422	−0.38 (−0.92, 0.16)	0.166	0.175
Control (n = 20)	62.5	(9.8)	63.0	(9.7)	0.54 (0.15, 0.93)	0.009			
BMI (kg/m^2^)									
Laughter yoga (n = 20)	23.4	(3.3)	23.5	(3.3)	0.06 (−0.11, 0.23)	0.455	−0.17 (−0.39, 0.06)	0.145	0.143
Control (n = 20)	24.9	(2.7)	25.2	(2.7)	0.23 (0.06, 0.39)	0.009			
Waist circumference (cm)									
Laughter yoga (n = 20)	87.4	(9.5)	86.6	(9.0)	−0.87 (−2.28, 0.55)	0.217	−1.10 (−3.23, 1.03)	0.302	0.133
Control (n = 20)	92.5	(8.1)	92.7	(8.0)	0.24 (−1.45, 1.92)	0.774			
Positive affect									
Laughter yoga (n = 19)	3.3	(1.7)	4.0	(1.2)	0.68 (0.13, 1.24)	0.019	0.68 (0.01, 1.36)	0.048	0.246
Control (n = 19)	4.0	(1.3)	4.0	(1.0)	0.00 (−0.43, 0.43)	1.000			
Negative affect									
Laughter yoga (n = 19)	2.3	(1.6)	2.3	(1.8)	0.05 (−0.60, 0.71)	0.867	−0.05 (−0.82, 0.71)	0.890	0.935
Control (n = 19)	1.9	(1.7)	2.0	(1.7)	0.11 (−0.35, 0.56)	0.630			
Subjective stress									
Laughter yoga (n = 18)	1.8	(0.7)	2.0	(1.0)	0.17 (−0.19, 0.52)	0.331	0.40 (−0.13, 0.93)	0.132	0.415
Control (n = 17)	2.2	(0.9)	1.9	(0.6)	−0.24 (−0.66, 0.19)	0.260			
Sleep duration (hours)									
Laughter yoga (n = 18)	6.0	(1.0)	6.4	(1.0)	0.39 (−0.04, 0.82)	0.074	0.47 (−0.06, 1.00)	0.080	0.058
Control (n = 18)	6.3	(1.4)	6.2	(1.5)	−0.08 (−0.43, 0.26)	0.616			

BMI, body mass index; HbA1c, hemoglobin A1c; SD, standard deviation; 95% CI, 95% confidence interval.

a P for comparing the difference in outcomes before and after the intervention using paired-samples t test.

b P for unadjusted between-group difference in changes in outcomes over 12 weeks using independent samples t test.

c P for adjusted between-group difference (adjustment for age, BMI and each dependent variable value at baseline) using analysis of covariance.

At baseline and 12-week follow-up, 14 (66.7%) and 19 (90.5%) individuals in the laughter yoga group, and 10 (47.6%) and 8 (38.1%) individuals in the control group reported having exercise at least twice a week, respectively. The number of individuals with exercise habits increased in the laughter yoga group but with no statistical significance. However, the adjusted between-group difference of HbA1c remained statistically significant after adding the change in exercise habits as a covariate. The mean change in HbA1c was 0% in the five participants who reported increased exercise habits and −0.32% in the 16 participants without exercise habit changes. Additionally, the number of individuals who reported eating until full and skipping breakfast was not significantly different between the two groups at baseline and 12-week follow-up (data not shown). The number of individuals who reported eating until full and skipping breakfast remained from baseline to 12-week follow-up in both groups.

## 4 Discussion

The results of this study showed that laughter yoga program for 12 weeks decreased HbA1c levels in individuals with type 2 diabetes. Additionally, the high attendance rate suggests that the program is feasible for the participants. To the best of our knowledge, this is the first randomized controlled trial that has evaluated the long-term effects of laughter yoga on glycemic control in individuals with type 2 diabetes.

The findings of the current study are consistent with previous studies demonstrating that laughter by watching a comedy show for 40 min (21) or a single 30-min session of laughter yoga (33) inhibits the increase in postprandial glucose levels in individuals with type 2 diabetes. The findings are also consistent with our previous study showing that the combination of laughter by watching comedy shows and exercise for 10 weeks decreased HbA1c levels among older adults (25).

The mean HbA1c levels changed from 7.07% to 6.82% in the laughter yoga group. Recent study has shown that maintaining HbA1c levels at <7% over 5 years is associated with significant reductions in the odds of being diagnosed with diabetes-related complications (3). Another study found that the secondary structure of hemoglobin in individuals with good glycemic control (HbA1c < 7.0%) was not significantly altered although elevated HbA1c levels contribute to hemoglobin structural modifications, which are associated with pathological complications in type 2 diabetes mellitus (42). A previous meta-analysis reported that every 1% increase in HbA1c is associated with a 15% increase in the hazard of all-cause mortality, a 25% increase in cardiovascular disease mortality, a 17% increase in cardiovascular diseases, and an 11% increase in stroke, and suggested a positive dose-response trend between HbA1c levels and cardiovascular outcomes in people with type 2 diabetes (5). A recent study in the USA also demonstrated that a 1% reduction in HbA1c is associated with a 13% reduction in diabetes-related total healthcare costs, resulting in an annual cost savings of $736 (43). Thus, changes in HbA1c in this study might be clinically significant in patients with type 2 diabetes.

A meta-analysis found that, in individuals with type 2 diabetes, low-to-moderate-intensity resistance exercise reduced HbA1c levels by 0.23%, and high-intensity resistance exercise reduced HbA1c levels by 0.61% (40). The 0.24% reduction in HbA1c levels in this study is similar to the effects of low-to-moderate-intensity resistance exercise. Another meta-analysis reported a 0.33% reduction in HbA1c levels in a psychological treatment group compared with a control group, and greater improvements in participants with higher baseline HbA1c levels (44). Similarly, a systematic review of healthcare interventions reported a 0.34% reduction in HbA1c levels, and subgroup analysis showed that populations with baseline HbA1c levels > 9.5% exhibited more reduction in HbA1c (0.58%) than populations with baseline HbA1c levels < 9.5% (0.17%) (45). Considering that the average baseline HbA1c level in our study was 7.1%, laughter yoga may be effective as an adjunctive therapy for the management of type 2 diabetes.

Although the mechanisms remain unclear, there are several possibilities for the effect of laughter yoga on diabetes. First, it has been reported that laughter upregulates genes related to natural killer cell activity in individuals with type 2 diabetes, which may ameliorate glucose intolerance (18). Second, laughter could influence glycemic control through the effects of positive affect that accompanies laughter. Positive psychological constructs such as positive affect, optimism and self-efficacy have been suggested to increase adherence to health behaviors (46), which may benefit individuals with type 2 diabetes. A longitudinal cohort study has also reported that positive affect such as enjoyment of life predicts lower risk of mortality in older adults with diabetes (47). In the present study, positive affect significantly increased from baseline to the 12-week follow-up in the laughter yoga group, which might be beneficial for glycemic control. In addition, laughter might have a stress-buffering effect. It has been reported that diabetes-related distress predicts poor glycemic control and poor medication adherence (48). Laughter yoga could attenuate cortisol stress response (30), which might buffer against the negative impact of stress. Third, increased energy expenditure during laughter might be beneficial for glycemic control. A study has suggested that 10–15 min of voiced laughter (by viewing a humorous film) could increase energy expenditure by 10–40 kcal (49). Laughter yoga may increase energy expenditure more than laughter during watching a humorous film, although no studies have examined energy expenditure during laughter yoga.

It has been reported that when people experience positive affect during a specific behavior, they are more likely to continue that behavior (14). The positive affect experienced through laughter yoga may have led to the high attendance rate in the laughter yoga program. Although most health behaviors are difficult to sustain, continuing laughter yoga as a habit may be relatively easy.

In this study, sleep duration tended to improve. This finding is consistent with previous studies reporting that laughter has favorable effects on sleep quality or insomnia. One study has shown that 1 h of laughter therapy once a week for 4 weeks improves insomnia and sleep quality among older individuals (50). Another study has reported that a 30-min laughter yoga session twice weekly for 8 weeks improves sleep quality in patients undergoing hemodialysis (51). Additionally, a significant correlation was observed between changes in sleep duration and HbA1c levels in the laughter yoga group (r = −0.47; *P* = 0.050). It has been reported that short sleep durations (less than 4.5–6 h/night) are associated with increased HbA1c levels in individuals with type 2 diabetes (52). Thus, the increase in sleep duration in the laughter yoga group might be associated with better glycemic control.

Laughter yoga combines simulated laughter with yoga breathing techniques. The effects of laughter and the effects of yogic breathing are difficult to distinguish because laughter also consists of mixed patterns of expiration, inspiration, and interval pauses (53). It has been reported that voiced laughter causes a 10%–20% increase in energy expenditure and heart rate compared with resting values (49), and may activate sympathetic activity (54). In contrast, most yogic breathing practices result in a parasympathetic shift of autonomic nervous system activity (55). Laughter may have effects similar to exercise, and yogic breathing may enhance the effects of relaxation. However, this study did not measure the heart rate. Further studies are needed to assess the effects of laughter yoga on the autonomic nervous system.

This study conducted a 12-week laughter yoga intervention. In a systematic review of the effects of laughter-inducing interventions, the duration of most laughter yoga interventions ranged 4 to 8 weeks and one study conducted a 12-week laughter yoga intervention (28). Another systematic review of the effects of laughter yoga in older adults reported that the duration of interventions ranged from 4 to 6 weeks (26). To our best knowledge, little is known about the effects of longer-term laughter yoga interventions and it remains unclear which duration of intervention is most effective. It is possible that longer-term intervention is more effective. On the other hand, a systematic review on yoga for type 2 diabetes reported that the duration of yoga intervention ranged one week to 26 weeks (a median of 12 weeks) (56). Additionally, a meta-analysis assessing the effects of yoga intervention on cardiovascular disease risk factors reported that the effects were most prominent in randomized controlled trials with 12 weeks of intervention duration, and fewer effects were found in shorter or longer interventions (36). More studies with various duration of laughter yoga intervention including especially longer-term interventions are needed to examine the most effective intervention duration.

In this study, we did not assess the sustainability of the effects of the intervention after the study period. Further follow-up studies are needed to examine how long the effects of laughter yoga are maintained. A meta-analysis showed that improved physical activity through behavioral change interventions is generally not sustained after the intervention (57). Motivating participants to continue laughter yoga beyond intervention termination would be important (e.g., introducing laughter yoga class in the local community or recommending making a new laughter yoga group).

This study has several limitations. First, the number of participants was small, and the study was conducted in a single center. Multicenter studies with larger sample sizes are needed to better understand the intervention’s efficacy and generalizability. The study participants were all Japanese living in an urban area and outpatients of the university hospital with relatively good glycemic control. Our findings may not apply to different populations, including other ethnic groups, those living in rural areas, and those with poorly controlled type 2 diabetes. Further studies are needed to evaluate the effects of laughter yoga on glycemic control in different populations. In contrast, the effects of laughter yoga on mental health have been reported worldwide (Asia, the Middle East, Australia, and United States) and in different clinical settings (28, 58). Therefore, this intervention could be applied to various populations. Second, changes in unknown psychosocial or lifestyle factors that were not assessed in this study might have affected glycemic outcomes. Additionally, group activity participation might have beneficial effects. Communicating with other participants and developing a sense of community could improve their motivation to attend the intervention. Third, diabetes-related distress was not assessed in this study. Further study is needed to assess distress using validated questionnaires. Fourth, positive and negative affect was measured using items from GDS-15. The Cronbach’s Alpha value in this study was acceptable for the positive affect items, however the value was relatively low for the negative affect items. The Cronbach’s Alpha value is influenced by the number of items, item inter-relatedness, and dimensionality, and a small number of items will underestimate the reliability (59). A study has suggested that a value of 0.50 is satisfactory when the items are fewer than 20 (60). However, using the ten items as one factor may not be suitable for this study population. Negative affect has various types. The factor structure of the GDS-15 has been reported to vary depending on the study population (38). For example, a study among community-dwelling older Japanese reported three factors: depressed mood, positive affect, and energy loss (61). Another study that included home-dwelling poststroke patients in Japan reported two factors: the positive affect and the depressed mood, including lack of energy (62). In addition, although the GDS-15 includes components of positive and negative affect, they might not represent pure positive and negative affect. Fifth, comorbid psychological conditions were not considered in the exclusion criteria. In terms of depression, no individuals received treatment for depression after confirming the medication status, and the baseline scores of the GDS-15 did not indicate depression in any participants. Sixth, sleep duration was self-reported, which might have affected the reliability of the data.

In conclusion, laughter yoga for 12 weeks decreased HbA1c levels in individuals with type 2 diabetes, and the program is feasible with a high attendance rate. The importance of psychological well-being in individuals with diabetes has been gradually recognized, and positive psychological interventions have been recommended (63). We propose that having fun could be a self-care intervention. Although further studies with larger numbers of participants are warranted to better evaluate the beneficial effects, laughter yoga may be applied as an easy, enjoyable, and effective option for self-managing type 2 diabetes.

## Data availability statement

The raw data supporting the conclusions of this article will be made available by the authors, without undue reservation.

## Ethics statement

The studies involving human participants were reviewed and approved by Scientific Ethics Committee of Fukushima Medical University. The patients/participants provided their written informed consent to participate in this study.

## Author contributions

MH analyzed and interpreted data, and wrote the manuscript. MH, TO, and YW conducted the study and contributed to data acquisition. TO, EE, KS, HIm, NF, HN, NK, IS, and HIs contributed to the concept and design of the study and participated in critical revision of the manuscript. HN, NK and IS recruited participants and interpreted the diabetes-related outcome measures. MH and TO are the guarantors of this work and, as such, had full access to all the data in the study and take responsibility for the integrity of the data and the accuracy of the data analysis. All authors contributed to the article and approved the submitted version.
